# Data on anti-insulation detection via Point of Thermal Inflexion (PTI) in 1248 cases; 13 climates, four occupancy profiles, six wall configurations and four insulation levels

**DOI:** 10.1016/j.dib.2017.04.016

**Published:** 2017-04-15

**Authors:** Yasin M. Idris, Masayuki Mae

**Affiliations:** Mae Laboratory, Department of Architecture, Graduate School of Engineering, The University of Tokyo, Tokyo 113-8656, Japan

**Keywords:** Thermal inflexion, Energy saving, Cooling set-point, Building envelope, Insulation optimisation, Thermal mass, Construction layer configuration, Overheating risk

## Abstract

The data in this article are the simulation results of 1248 cases that were carried out to detect anti-insulation behaviour in the article titled “Anti-insulation mitigation by altering the envelope layers’ configuration” (Idris and Mae, 2017) [1]. These cases are generated by a matrix of 13 climates, 6 envelope layer configurations, 4 occupancy profiles and 4 levels of insulation thickness. The data are concerned with the annual cooling and heating loads of these cases. In addition, the data include the Point of Thermal Inflexion (PTI) values and their anti-insulation pattern, when PTI is found. The PTI values are compiled in a single summary file and supplied as well. All These data are shared via this article where they can be reused in different ways, but mainly for serving researchers that intend to approach anti-insulation behaviour from different points of view.

**Specifications Table**

**Table:** 

Subject area	*Energy in Buildings*
More specific subject area	*Cooling energy conservation*
Type of data	*Excel Files*
How data was acquired	*The data are the annual cooling and heating load simulation results. The cooling loads were further organised to derive the anti-insulation patterns and find their Points of Thermal Inflexion (PTI) values.*
Data format	*.xls*
Experimental factors	*The PTI value is sometimes modified based upon the anti-insulation pattern of the case.*
Experimental features	*Data were produced by simulating the thermal loads of a windowless single-cell room using EnergyPlus over a matrix of 1248 cases.*
Data source location	
Data accessibility	*Data is within this article*

**Value of the data**

• The data of such a vast number of simulation cases would promote better understanding of anti-insulation behaviour by allowing observation of it under various groups of parameters (variables clusters).
• These data can also be of great importance and time saving for studies that are aimed at developing the governing correlation equations or to find weights of the anti-insulation influencing factors.
• The data involved six layer configurations, in which studies concerned with the dynamic thermal behaviour of the wall configurations would find it useful. Specifically, in studying the configurations performance over two principal parameters, i.e. over 13 climates and the two AC operation profiles (continuous and intermittent).
• By providing the heating loads, further studies can utilise the annual total loads (Sum of cooling and heating) to systematically develop a novel insulation optimisation approach, which is solely based on anti-insulation.

## 1 Data

This article dataset is comprised of 53 excel files. The first file, named “PTI Summary of All 1248 cases”, is the PTI values of all the 1248 cases which are colour-coded based on their anti-insulation patterns. This file has six tabs, four of these are denoting the data summary of the four occupancy profiles. The other two tabs are for the cases grand summaries, i.e. the PTI values compilation table, and the PTI patterns summary table.

The subsequent 52 files are for the specific environmental conditions, under which the six layer configurations are examined. The 52 files are the production of 13 climates and four occupancy schedule profiles. The file naming convention is as follows [Serial Number_Climate Representative City_Occupancy Profile.xls], and the naming abbreviations are provided in Table 1. Each file of these 52 files has 8 tabs; six are the primary PTI graphs for the 6 layer configurations. The remaining two tabs contain; first, a tab that comprises the raw cooling and heating loads simulation results, and second, a tab that displays the layer configurations performance ranking.

## 2 Experimental design, materials and methods

The EnergyPlus standalone version (8.4) was employed as a simulation software. The “IdealLoadsAirSystem” object was used to calculate the annual cooling and heating loads, where It calculates the energy that is being consumed to maintain the desired set-points. A window-less single-zone room of 6×6×3 m served as a case study, and It was assumed that all its surfaces (walls, roof and floor) have the same construction. The six construction configurations were produced by rearranging brick and insulation board layers. The total brick width was fixed at 20 cm across the configurations, whereas the insulation varied to generate the PTI graphs. For each of the 1248 cases, the PTI values were obtained by plotting the cooling load of 25 permutations, i.e. five insulation thicknesses and five cooling set-points, while having the 20 cm bare-brick cases as the energy saving/loss benchmarks. Eventually, the PTI values are sorted and statistically processed, then transferred into the spreadsheets that are supplied herewith in this article. Further information on the adopted methodology is presented in [1].

## Figures and Tables

**Table 1:** The 52 environmental files naming convention.

Code1	Representative City	Code2	Occupancy Profile
Miam	Miami, FL	Res	Residential
Rydh	Riyadh - KSA	Ofc	Office
Hstn	Houston, TX	ResConL	Residential with Continuous Load
Phnx	Phoenix, AZ	OfcConL	Office with Continuous Load
Mphs	Memphis, TN		
Epso	El Paso, TX		
SFrn	San Francisco, CA		
Bltm	Baltimore, MD		
Albq	Albuquerque, NM		
Salm	Salem, OR		
Cago	Chicago, IL		
Bois	Boise, ID		
Vcvr	Vancouver, BC- CA		
